# Deficiency in Androgen Receptor Aggravates Traumatic Brain Injury-Induced Pathophysiology and Motor Deficits in Mice

**DOI:** 10.3390/molecules26206250

**Published:** 2021-10-15

**Authors:** Yu-Hsin Chen, Yen-Chou Chen, Ling-Ling Hwang, Liang-Yo Yang, Dah-Yuu Lu

**Affiliations:** 1 Graduate Institute of Medical Sciences, College of Medicine, Taipei Medical University, Taipei 11031, Taiwan; amytokimo@hotmail.com (Y.-H.C.); yc3270@tmu.edu.tw (Y.-C.C.); llhwang@tmu.edu.tw (L.-L.H.); 2 Department of Physiology, School of Medicine, College of Medicine, China Medical University, Taichung 40402, Taiwan; 3 Laboratory of Neural Repair, Department of Medical Research, China Medical University Hospital, Taichung 40447, Taiwan; 4 Department of Pharmacology, School of Medicine, China Medical University, Taichung 404333, Taiwan; 5 Department of Photonics and Communication Engineering, Asia University, Taichung 404333, Taiwan

**Keywords:** traumatic brain injury, controlled cortical impact, androgen receptor, ARKO mice, motor activity

## Abstract

Androgens have been shown to have a beneficial effect on brain injury and lower reactive astrocyte expression after TBI. Androgen receptors (ARs) are known to mediate the neuroprotective effects of androgens. However, whether ARs play a crucial role in TBI remains unknown. In this study, we investigated the role of ARs in TBI pathophysiology, using AR knockout (ARKO) mice. We used the controlled cortical impact model to produce primary and mechanical brain injuries and assessed motor function and brain-lesion volume. In addition, the AR knockout effects on necrosis and autophagy were evaluated after TBI. AR knockout significantly increased TBI-induced expression of the necrosis marker alpha-II-spectrin breakdown product 150 and astrogliosis marker glial fibrillary acidic protein. In addition, the TBI-induced astrogliosis increase in ARKO mice lasted for three weeks after a TBI. The autophagy marker Beclin-1 was also enhanced in ARKO mice compared with wild-type mice after TBI. Our results also indicated that ARKO mice showed a more unsatisfactory performance than wild-type mice in a motor function test following TBI. Further, they were observed to have more severe lesions than wild-type mice after injury. These findings strongly suggest that ARs play a role in TBI.

## 1. Introduction

Traumatic brain injury (TBI) is a severe global health problem in more than 10 million people every year, affecting over 10 billion people worldwide [1,2]. TBI often causes irreversible neurological, motor, and cognitive impairment [3,4,5]. Patients and their families who suffer from TBI experience long-lasting problems and challenges in their daily lives [6]. Severe TBI induces skull fracture, hemorrhage, and vascular injury, which may often occur alone or in different combinations [7]. Secondary TBI symptoms present shortly following the primary injury and can last for hours or days. The symptoms include impairment of the microvasculature; inflammation, damage, and dysfunction of glial cells; and loss of neuronal cells that further affect behavioral functions [8,9]. Therefore, it is pivotal to further understand TBI’s pathophysiology before we can develop better therapeutic agents or strategies to treat it.

TBI is a complex disease caused by primary injury, followed by complex secondary damage with increasing astrogliosis and activation of many proteases, such as activation of caspases and calpain-II [10,11,12]. A recent study showing the inhibition of calpain-II by genetic or pharmacological treatment in mice indicated that calpain-II is a potential therapeutic target in TBI and repeated concussions [13]. Glial fibrillary acidic protein (GFAP) is abundant in astrocytes and is necessary for CNS integrity and maintenance. A recent analysis of clinical reports has suggested that serum GFAP can be a useful tool to assess the clinical outcomes of patients with aneurysmal subarachnoid hemorrhage (SAH) [14]. GFAP is also a substrate of calpain-II protease and a widely used biomarker of astrogliosis in TBI [15]. In addition, alpha-II- spectrin, a neuronal scaffolding protein, is also a substrate of calpain II [16]. In particular, a recent clinical study reported correlation of alpha-II-spectrin breakdown product (SBDP) level with the clinical outcome and severity in patients with aneurysmal SAH [14]. Accumulation of breakdown products (BDPs) from GFAP and alpha-II-spectrin cleaved by calpain-II has been identified in the cerebrospinal fluid (CSF) after TBI [17]. Studies have demonstrated that SBDPs appear to be a useful diagnostic measure of TBI severity and outcome [18,19]. TBI results in chronic changes in the brain levels of GFAP and alpha-II-spectrin, and SBDP150 may act as an acute-chronic biomarker [20]. Recently, GFAP and alpha-II-spectrin breakdown products also have been considered useful biomarkers in the diagnosis and prognosis of TBI [21].

Androgen receptors (ARs) have been reported to play essential roles in the regulation of the male reproductive system development and other biological functions [22]. In particular, they have also been reported to exert critical physiological regulation on the development and maintenance of the nervous system [23]. Furthermore, ARs have been reported to be expressed in the central nervous system (CNS), including neurons, glial cells, cerebral endothelium, and smooth muscle cells [24,25,26]. Importantly, androgens have been found to increase anti-anxiety behavior, enhance cognitive performance [27], and induce neuroprotective effects directly through the ARs present in the human brain neurons [28]. Evidence shows that androgens protect neurons against the neurotoxicity of amyloid-beta peptides and cognitive dysfunction via ARs [29,30]. In addition, androgens can attenuate the axotomy-induced loss of developing facial motor neurons [31]. It has been reported that TBI patients with lower testosterone levels tend to stay in the hospital longer and show lower functional independence measure scores [32]. Female sex hormones, progesterone, and estrogen have been recently characterized as important contributors to TBI responses and outcomes [33]. However, the neuroprotective effects of the androgen receptor in TBI remain unclear.

Autophagy is one of the most critical factors in the pathogenesis of many CNS diseases [34,35]. Autophagy dysfunction due to reduced lysosomal function and consequent autophagosome accumulation has been observed in various neurodegenerative diseases, including Alzheimer’s disease (AD) [36], Parkinson’s disease (PD) [37], and Huntington disease (HD) [38]. Furthermore, accumulating evidence shows autophagy activation in injured brains [39,40,41]. Beclin-1 is involved in autophagy regulation and functions in tumorigenesis, and neurodegeneration [42]. Autophagy occurs after experimental TBI, which frequently presents autophagosomal vacuoles and secondary lysosomes in the brain [43]. The autophagy marker Beclin-1 increases after TBI and is reported to be involved in injury-induced cell death at the injured site. However, autophagy inhibition improves behavioral outcomes and reduces lesion volume after TBI [44,45].

Dr. Chang’s group has generated the androgen receptor knockout (ARKO) mice by using a cre-lox conditional knockout strategy [46]. The ARKO mice provide an animal model to study androgen functions in pathophysiology, and suggests AR may be a target for therapy in the future [47]. The aims of this study were to investigate the regulatory functions of ARs and the potential role of biomarkers in TBI. We hypothesized that AR knockout aggravates the brain lesion volume and motor function deficits induced by TBI, and subsequently affects necrosis, autophagy, and neuroinflammation. Understanding TBI pathophysiology will facilitate the advancement of novel therapeutic strategies for treating or alleviating this disease.

## 2. Results

### 2.1. Effects of Androgen Receptor Knockout on SBDP150 Expression in Mice following TBI

**Figure 1:**
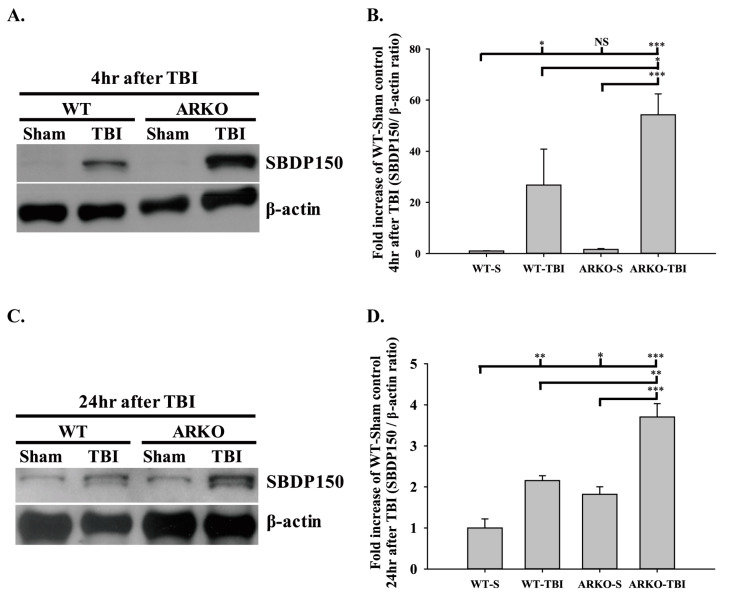
Androgen receptor knockout enhances traumatic brain injury (TBI)-induced necrosis marker spectrin breakdown product 150 (SBDP150) expression after brain injury in mice. Protein levels of the necrosis marker SBDP150 were examined in the wild-type (WT) and androgen receptor knock out (ARKO) mice brain tissue after TBI, using Western blot technology. SBDP150 expression (**A**) and the quantitative level (**B**) at 4 h following TBI. TBI enhanced SBDP150 expression of the injured WT brain compared with the WT sham. SBDP150 levels increased significantly in ARKO mice following brain injury than in ARKO sham and WT mice with TBI. (**C**) ARKO mice showed a significantly higher level of SBDP150 expression than WT mice 24 h after TBI. (**D**) Quantitative level of SBDP150 expression at 24 h after TBI. All data are presented as the mean ± standard error. NS, no significant difference; * *p* < 0.05, ** *p* < 0.01, *** *p* < 0.001; *n* = 3 in each group.

SBDP150 is an alpha-II-spectrin breakdown product cleaved by calpain cysteine proteases and is produced in necrotic cells [48,49]. It is a potentially reliable biomarker for severe TBI in animals and humans [50,51]. We first tested whether knockout of the androgen receptor affects the expression of the necrosis marker SBDP150 following TBI. We assessed SBDP150 levels in the WT and ARKO littermate brain tissue at 4 h and 24 h after TBI. Our results indicate that TBI significantly increased SBDP150 expression in the WT mice at 4 h (F [3,8] = 15.078; *p* < 0.05) and 24 h after TBI (F [3,8] = 25.317; *p* < 0.01) compared with the sham control group. No significant differences were found between the WT sham and ARKO sham groups after injury. Further, knockout of androgen receptor enhanced the expression of SBDP150 at 4 h after TBI compared with that in WT mice (*p* < 0.05) (Figure 1A,B). At 24 h after TBI, ARKO mice showed a much higher expression of SBDP150 than WT mice (*p* < 0.01) (Figure 1C,D). Our results showed that the expression of SBDP150 increased following TBI, and knockout of androgen receptor significantly enhanced TBI-induced SBDP150 expression. The present study suggests that androgen receptor knockout enhances necrosis in TBI.

### 2.2. Effects of Androgen Receptor Knockout on GFAP Expression in Mice following TBI

**Figure 2:**
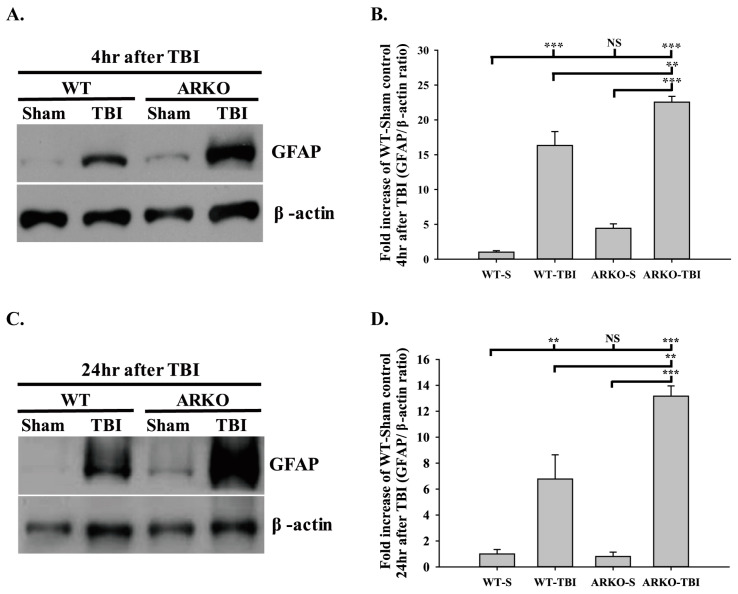
Androgen receptor knockout promoting GFAP expression induced by TBI GFAP expression was evaluated by Western blot. (**A**) Androgen receptor knockout induces GFAP expression at 4 h after TBI. (**B**) The quantitative data of GFAP level at 4 h following brain injury. (**C**) ARKO mice show TBI-induced GFAP expression enhancement compared with WT 24 h after TBI. (**D**) Quantitative data of GFAP level at 24 h following TBI. All data are presented as the mean ± standard error. NS, no significant difference; ** *p* < 0.01, and *** *p* < 0.001; *n* = 3 in each group.

**Figure 3:**
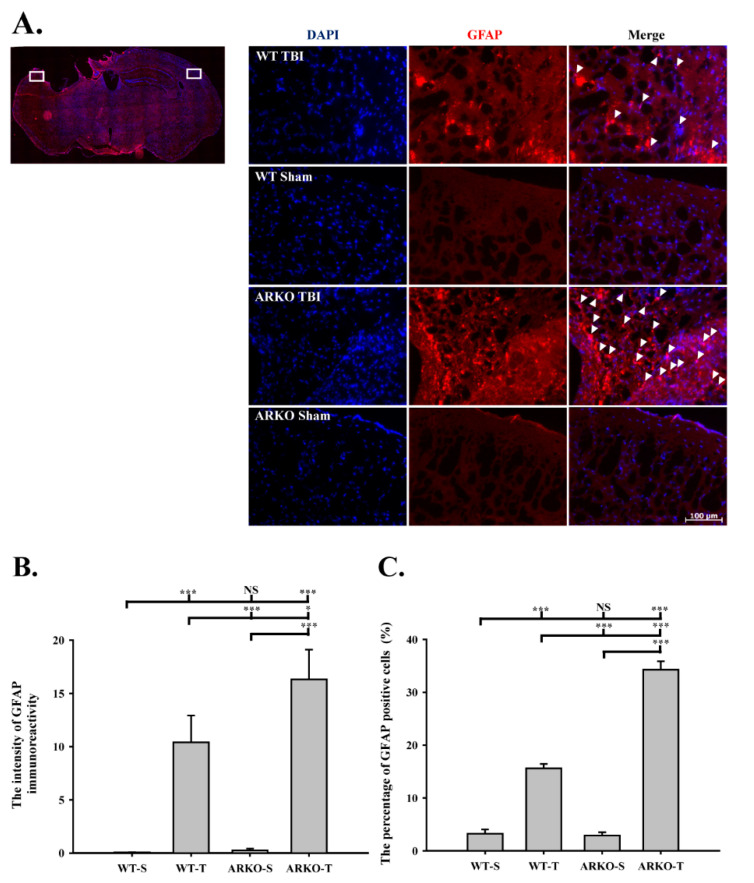
Androgen receptor knockout increases the TBI-induced GFAP expression around the cortical injury site. (**A**) Illustrations of the regions of interest (white areas) of the mice brain after TBI are shown in left panel. WT and ARKO mice were performed with TBI or sham, and then stained with immunofluorescence of GFAP. The GFAP positive cells were indicated by white arrowhead. ARKO mice showed the increasing cells of GFAP expression. Blue color, DAPI (4′,6-diamidino-2-phenylindole); red color, GFAP. (Images: x200 magnification of the ipsilateral and the contralateral hemispheres; scale bar = 100 μm) (**B**) The intensity of GFAP immunoreactive level with normalized intensity fluorescence unit in the four experimental groups is presented. (**C**) The percentage of GFAP positive cells counterstained with DAPI in the four experimental groups is presented. The expression of GFAP at the cortical injury site was calculated from six different bregma levels. WT-S, wild-type sham; WT-T, wild-type with TBI; ARKO-S, ARKO sham; ARKO-T, ARKO with TBI. All data are presented as the mean ± standard error. NS, no significant difference; * *p* < 0.05, and *** *p* < 0.001; *n* = 7 in each group.

We further evaluated whether knockout of the androgen receptor influences astrogliosis after TBI. As shown in Figure 2A,B, we found that the GFAP expression was upregulated in the ARKO mice 4 h after TBI compared with the WT mice (F [3,8] = 78.498; *p* < 0.01). In parallel, TBI-induced GFAP expression in the ARKO mice at 24 h was significantly increased than that in the WT mice (F [3,8] = 31.638; *p* < 0.01) (Figure 2C,D). There was no significant difference in GFAP expression between the WT and ARKO mice without brain injury. To further examine whether knockout of the androgen receptor affects astrogliosis in the long term after TBI, we evaluated the GFAP level at 21 days after TBI using immunofluorescence. As shown in Figure 3A, GFAP-positive cells were observed around the cortical injury site on the injured hemisphere at 21 days following TBI in both the WT and ARKO mice. Moreover, TBI-induced GFAP upregulation in the ipsilateral cortex was observed in both the WT (F [3,24] = 18.077; *p* < 0.001) and ARKO mice (*p* < 0.001) compared with the sham control (Figure 3B). Meanwhile, the number of GFAP positive cells was elevated following TBI in WT (F [3,24] = 205.134; *p* < 0.001) and ARKO mice (*p* < 0.001) compared with sham animals (Figure 3C). There was no statistically significant difference in GFAP expression between the WT sham and ARKO sham controls. GFAP upregulation was also observed in ARKO mice compared with that in the WT (*p* < 0.001). Our results revealed that GFAP expression was increased after TBI in both WT and ARKO mice. Together, these results indicate that androgen receptor knockout aggravates the TBI-induced astrogliosis effect in the injured cortex, and this effect was long-lasting (for over three weeks after TBI).

### 2.3. Effects of Androgen Receptor Knockout on Beclin-1 Expression in Mice following TBI

**Figure 4:**
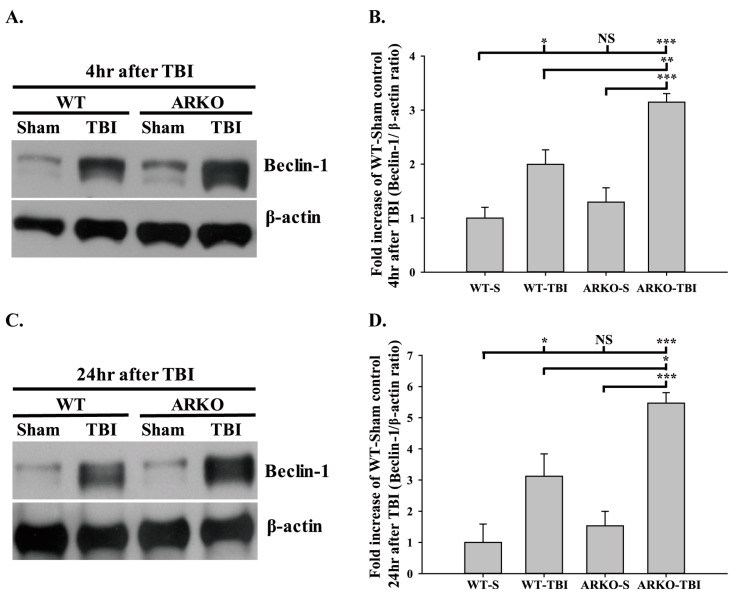
Androgen receptor knockout increases the TBI-induced Beclin-1 expression after brain injury. (**A**) TBI increases Beclin-1 expression. However, knockout of androgen receptor further increases Beclin-1 expression 4 h after injury. (**B**) Quantitative level of Beclin-1 expression at 4 h following TBI. (**C**) Beclin-1 expression shows a significant increase in ARKO mice, compared with WT mice in 24 h following TBI. (**D**) Quantitative level of Beclin-1 expression at 24 h following TBI. All data are presented as the mean ± standard error. NS, no significant difference; * *p* < 0.05, ** *p* < 0.01, and *** *p* < 0.001; *n* = 3 in each group.

Since autophagy plays a remarkable role in brain injury, we evaluated whether the androgen receptor is involved in TBI-associated brain injury and autophagy. Figure 4A and 4B show that knockout of the androgen receptor further enhanced the TBI-induced Beclin-1 expression at 4 h (F [3,8] = 17.508; *p* < 0.05) after TBI compared with the WT (*p* < 0.01). Similarly, knockout of the androgen receptor significantly increased the TBI-induced Beclin-1 expression at 24 h after TBI compared with the WT (F [3,8] = 13.510; *p* < 0.05) (Figure 4C,D). There were no significant differences between the WT sham and ARKO sham groups at both 4 and 24 h. The present study showed that androgen receptor knockout promotes TBI-induced autophagy marker Beclin-1 expression in injured brain tissue. These results demonstrate that the androgen receptor is involved in regulating autophagy following TBI.

### 2.4. Androgen Receptor Knockout Affects the Motor Behavioral Outcomes and Lesion Volumes in Mice following TBI

**Figure 5:**
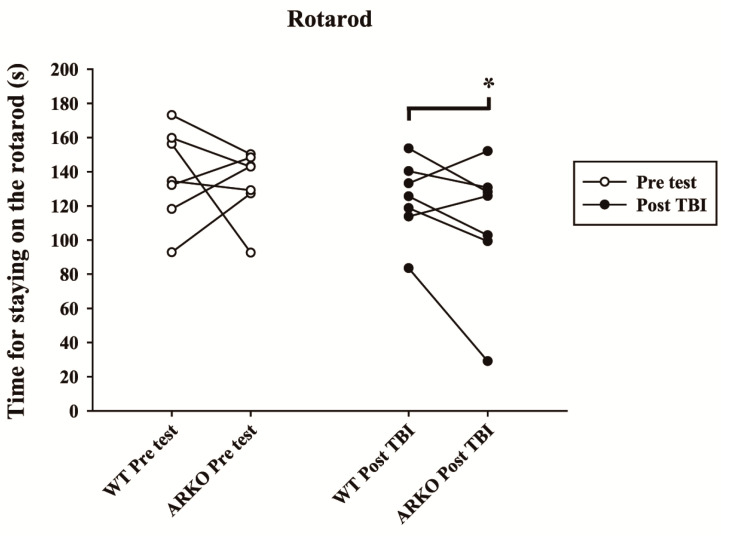
Androgen receptor knockout significantly decreases the motor function of mice after TBI. The time during which the mice stayed on the rod at an accelerating speed was evaluated before (pre) and 20 days after TBI (post). The white circles represent the rotarod test before TBI, and the black circles represent rotarod results in the WT and ARKO group after TBI. Each circle shows the mean of five trials of the rotarod test. The data points of each pair of littermates are connected by a line. Compared with the WT, the time spent on the rod shows a significant difference in the ARKO mice following TBI. All data are presented as the mean with paired *t*-test; * *p* < 0.05 versus wild-type; *n* = 7 in each group.

**Figure 6:**
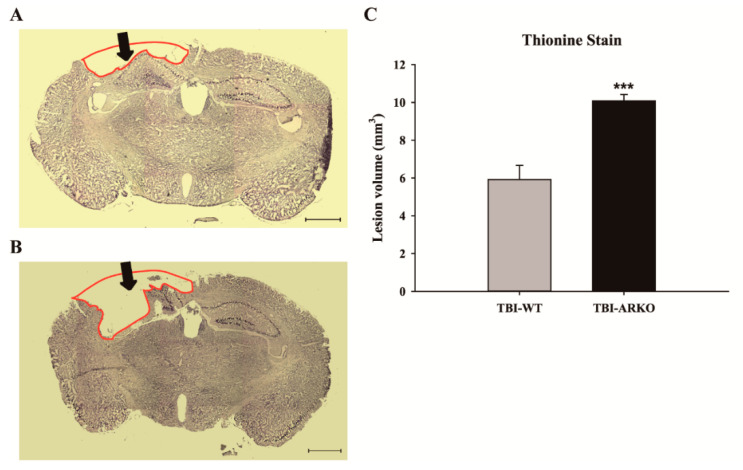
Androgen receptor deleted mice show a larger brain lesion volume than the WT following TBI. Thionine stained section of WT mice (**A**) and ARKO mice (**B**) brain after TBI. The black arrows indicate the impact site of TBI, 21 days following the injury. Red lines illustrate the lesioned area. (Images: x25 magnification, scale bar = 1 mm) (**C**) The total brain lesion volume is calculated. Compared with the WT impacted with TBI, androgen receptor knockout significantly increases the lesion volume in ARKO mice. All data are presented as the mean ± standard error; *** *p* < 0.001 versus wild-type; *n* = 7 in each group.

To elucidate whether knockout of the androgen receptor influences animal behavioral outcomes, we used the rotarod task to evaluate the motor function of paired experimental mice before and after injury. The results showed that androgen receptor knockout mice spent significantly less time on the rotarod at an accelerating speed, 20 days after TBI (Figure 5). In contrast, there was no statistically significant difference in rotarod performance between the WT and AKRO mice littermates without brain injury (t = 0.372, df = 6). Motor function in ARKO mice was significantly reduced compared with their paired WT littermates 20 days after TBI (t = 2.515; df = 6; *p* < 0.05). Further, to understand whether AR knockouts enhance TBI-induced lesion, the total volumes of brain lesions following TBI were evaluated. After the rotarod behavioral test, mice were sacrificed at 21 days following TBI and perfused for histological analysis. Thionine staining was performed to analyze neuronal degeneration. As shown in Figure 6A and B, ARKO mice showed a larger brain lesion volume than the WT following TBI (F [1,12] = 25.72; *p* < 0.001) (Figure 6C). Our results indicate that knockout of the androgen receptor aggravates TBI-induced motor deficits and enhances TBI-injured brain lesion volume.

## 3. Discussion

Aberrant androgen receptors (ARs) activity, which usually occurs with mutations or binding partner misregulation, can be clinically recognized as androgen insensitivity syndrome and prostate cancer [52]. ARs have been reported to regulate the hypothalamic–pituitary–gonadal axis and reproductive behaviors to modulate cognition, anxiety, and other non-reproductive functions in the CNS [25]. Accumulating evidence suggests that many endocrine hormones play regulatory roles in the pathophysiology of brain injury [53,54,55]. A recent report also suggested that androgens exert preventive and therapeutic effects on various neurodegenerative diseases, such as AD, PD, multiple sclerosis (MS), and amyotrophic lateral sclerosis (ALS) [56]. Androgen receptor signaling has also been found to modulate hippocampal neurogenesis in a rodent model study [57]. Importantly, androgenic signaling has also been suggested to support the blood–testis barrier (BTB) formation and integrity in selective deletion of ARs in mouse Sertoli cells [58]. Androgens have also been recognized as remyelinating agents, and the brain AR may be a promising drug target for remyelination therapy [59]. In particular, Ars have been found that regulate neuronal activity by acting as a transcriptional regulator to modulate the expression of many target genes [60]. By using a cre-lox conditional knockout strategy, Dr. Chang’s group has generated the ARKO mice [46], which provide an animal model to study androgen functions in pathophysiology [47]. In the current study, we demonstrated for the first time that knockout of the androgen receptor enhances TBI-induced SBDP150, Beclin-1, and GFAP, which are related to necrosis, autophagy, and astrogliosis, respectively. We considered a reasonable experimental time point for this study to clarify the effects of androgen receptor knockout on protein expression of the biomarkers. We analyzed the acute phase influence at 4 and 24 h in our study, generally used to test experimental proteins. Our experimental design considered using the appropriate, usual checkpoint for SBDP150, Beclin-1, and GFAP proteins to observe the better result of the behavioral test and the severity of TBI measurement. Motor function deficits that occurred after brain injury were recognized from one month to eight weeks in adult mice [61,62]. Gender could be an important factor in the performance of motor function in rotarod test of cortical injury impairment [63]. The rotarod task has been recognized to possess greater sensitivity than other behavioral tests, such as beam-balance and beam-walking tasks, to evaluate motor deficits following TBI [64]. Our results support the previous findings that the brain lesion volume increases in ARKO and wild-type mice following TBI. In addition, knockout of the androgen receptor worsens motor function after TBI. These results provide new insights into the neuroprotection of ARs in the regulation of pathological outcomes and motor deficits following TBI.

Upregulation of proteases such as calpains and caspases constitutes the protease-substrate pathways related to TBI and neurodegeneration [65]. The spectrin protein expression can be analyzed in various glial cells, including oligodendrocytes, astrocytes, and Schwann cells in the central and peripheral nervous systems [66]. It has also been suggested that CSF SBDP levels can predict injury severity and mortality after severe TBI and can complement the clinical assessment [18]. Clinically, SBDP150 and not SBDP 120, both markers of calpain proteolysis by caspase-3, have been reported to increase significantly in the CSF of patients with TBI [50]. In addition, SBDP150 was also found to be significantly greater in patients with worse scores on the Glasgow Coma Scale (GCS) at 24 h after TBI compared with those whose GCS scores were better at the same time-point [50]. Further, a previous study has suggested that, in contrast to other spectrin proteins, such as SBDP120, SBDP150 may act as a prominent diagnostic biomarker in the acute phase of TBI [67]. CSF levels of SBDP150 have been found to be elevated in most brain regions and in the CSF of patients with severe TBI [18]. The SBDP150 expression could be detected from 2 to 72 h post-injury [68,69,70]. In the present study, the ARKO mice and control littermates had a higher rate of SBDP150 cleavage than observed in patients with TBI. Our results support the previous studies that experimental TBI can elevate alpha-II-spectrin cleavage product–SBDP150 levels in brain regions associated with caspase- or calpain-dependent cell death processes [71,72]. Furthermore, our data revealed that androgen receptor knockout enhances SBDP cleavage in TBI. Based on this evidence, we suggest that ARs may play a protective role against necrotic death of neurons.

The GFAP biomarker expression is highly correlated with neurologic function, brain lesion, and behavioral outcomes [73]. A recent clinical study reported a direct association between GFAP in serum and clinical variables and the severity of neurologic complications in critically ill children [74]. Astrogliosis is not necessarily a maladaptive process and may provide benefits following TBI [15]. Since the characteristic of the TBI biomarker GFAP was assessed in the neurophysiology of glia [75,76,77], our findings extend the importance of the androgen receptor in regulating GFAP activity in the context of TBI. In a clinical brain-injury treatment trial, plasma GFAP levels were significantly increased in patients with TBI from day 0 up to day 90 [78]. In a recent preclinical study of TBI, the serum levels of both GFAP and SBDP150 were used as biomarkers for predicting brain-cell death [79]. The present study supports the previous finding that the GFAP level increase correlates with behavioral and morphological changes [80]. Recently, it has been found that androgens protect the neurons from anti-inflammatory effects and astrocytes through activating AR [56]. Previous studies have demonstrated that testosterone reduces reactive astrocytes in peripheral motor neurons [81] and brain injury [82]. ARs are generally thought to mediate the effects of androgens, and the expression of ARs is induced in glial cells following brain injury [83]. Our results show that AR knock out further increased the expression of GFAP, which is TBI-induced, around the injury site.

Beclin-1 is one of the critical molecules of the autophagosome complex, which recruits many autophagy-related proteins to the phagophore membrane in the process of autophagosome formation [84]. The autophagy marker Beclin-1 rapidly increases near the injury site from 4 h and lasts for at least three weeks after cortical injury [40,85]. Furthermore, downregulation of the expression of the crucial autophagic protein Beclin-1 has been shown to reduce neurological severity in TBI [86,87]. Numerous reports have demonstrated that inhibiting neuronal autophagy and neuroinflammatory responses protects against TBI [88,89]. In hypoxia-ischemia-induced brain injury, Beclin-1 has been observed to co-localize with neurons but not GFAP-positive cells [90]. A recent study has also reported that hypothalamic astrocyte autophagy regulates obesity and systemic metabolism [91]. The antidepressant fluoxetine has also been suggested to promote autophagic flux and enhance autophagosome fusion in astrocytes [92]. Further, upregulation of autophagy flux in astrocytes has been suggested to contribute to endogenous neuroprotective and neuro-recovery effects after stroke [93]. Our results support previous studies showing that autophagy has a detrimental role in brain injury and that the TBI-induced autophagy effect may be regulated through the androgen receptor. However, more studies are needed to understand the regulation of ARs in neuronal and astrocyte autophagy after TBI.

## 4. Materials and Methods

### 4.1. Animal Model

Adult male androgen receptor knockout (ARKO) mice and wild-type male (WT) littermates were used in this study. Breeding pairs of heterozygous female (fAR/AR) ACTB Cre (-) and male (AR/Y) ACTB Cre (+) mice were generously provided by Dr. Chawn-Shang Chang at the University of Rochester, USA. The heterozygous female (fAR/AR) ACTB Cre (-) mice carrying the homozygous floxed *AR* gene were mated with male (AR/Y) ACTB Cre (+) mice to generate WT male mice [ARWT (AR/Y) ACTB Cre (+)] and male mice with androgen receptor knockout [ARKO (ar/Y) ACTB Cre (+)], which were used in the present study. Animals were housed on a 12-h light/dark cycle (lights on at 7:00 a.m.) and received ad libitum access to food and water. Animal protocols were approved by the Institutional Animal Care and Use Committee (IACUC; LAC-2014-0379). All animal procedures were performed in compliance with the National Institutes of Health Guidelines for the Care and Use of Laboratory Animals.

### 4.2. Experimental Design and Procedures

Thirteen pairs of ARWT: (AR/Y) ACTB Cre (+) and ARKO: (ar/Y) ACTB Cre (+) adult male littermate mice were used in this study. Mice littermates were anesthetized by intraperitoneal injections of Zoletil 50 (Tiletamine hydrochloride and Zolazepam hydrochloride, 25 mg 0.5 mL^−1^ kg^−1^, VIRBAC Laboratories, Carros, France) and Rompun (xylazine, 10 mg 0.5 mL^−1^ kg^−1^, Bayer AG, Leverkusen, Germany). The mice underwent a craniotomy at the left parietotemporal cortex, using a controlled cortical brain injury device. Paired littermates were randomly grouped for different time-course experiments or behavioral tests after TBI. Six paired littermates received TBI, three pairs were sacrificed 4 h after TBI, and the other three pairs were sacrificed 24 h after TBI for Western blotting to analyze the expression levels of the necrosis marker SBDP150, autophagy-related protein Beclin-1, and injury marker GFAP in the brain. The motor function of the other seven pairs of littermates was evaluated by rotarod behavioral tests 20 days after TBI and then sacrificed at 21 days for histological analysis.

### 4.3. Genotyping

Genomic DNA was extracted from mice tails, using a modified phenol/chloroform extraction method, as previously described [94]. The tail was placed in a solution of sodium dodecyl sulfate and proteinase K at 45–55 °C, overnight. The supernatant was mixed with phenol:chloroform:isoamyl alcohol (25:24:1) and then centrifuged. The obtained supernatant was mixed with chloroform and centrifuged to extract DNA. The genomic DNA was mixed with 100% ethanol at −20 °C, overnight, for precipitation. After centrifugation, the DNA was dried, dissolved in ddH_2_O, and stored at −20 °C.

### 4.4. Polymerase Chain Reaction

The purity of the isolated DNA was estimated by optical density evaluation for polymerase chain reaction (PCR). After PCR was completed, each sample was subjected to electrophoresis on a 2% agarose gel at 100 V for approximately one hour in TAE buffer. Heterozygous female (fAR/AR) ACTB Cre (-) mice crossed with male (AR/Y) ACTB Cre (+) mice to generate ARKO mice may have four possible genotypes: (ar/Y) ACTB/Cre (+), (ar/AR) ACTB/Cre (+), (AR/Y) ACTB/Cre (+), and (AR/AR) ACTB/Cre (+). We used the following primers: “select” (5′-GTTGATACCTTAACCTCTGC-3′), exon “2–3” (5′-TTCAGCGGCTCTTTTGAAG-3′), and exon “2–9” (5′-CCTACATGTACTGTGAGAGG-3′) to identify the mice genotype. Sequence “select” is located in intron 1, and the 3′ end primer “2–9” is located in intron 2. ARKO, WT, and floxed AR PCR products were 238, 594, and 800 bp in size. Primer exon “2–3” was used to detect the floxed AR on the X chromosome that amplified a product of 460 bp for examining the WT allele. We investigated the expression of Cre, the sex-determining region of the Y-chromosome (Sry), and interleukin 2 (IL-2) as internal controls for the genotyping PCR. PCR conditions and primer design were based on the Jackson Laboratory protocols.

### 4.5. Controlled Cortical Impact

TBI was induced by a controlled cortical impactor (CCI), TBI-0300 (1 mm impact depth, 5 m.s^−1^ impact velocity, and 500 ms dwell time) (Precision Systems and Instrumentation, LLC, Fairfax, VA, USA). As mentioned in the experimental design and procedures, male mice littermate brains were exposed after anesthesia. The exposed brain underwent a craniotomy at the left parietotemporal cortex. A 3 mm diameter impact was then made to the head centered 3 mm posterior to the bregma and 3 mm lateral to the midline. Cortical brain injury was induced by the impactor directly affecting the brain surface. Post-injury, the mouse skull was closed, and the skin was sutured immediately.

### 4.6. Western Blot

Mice were sacrificed 4 and 24 h after CCI-induced TBI, and the brains were removed. Each brain was separated into two parts: the lesioned hemisphere and the contralateral intact hemisphere. Brain tissue was collected and stored separately in liquid nitrogen. Proteins were extracted from the injured cerebral hemisphere and the intact contralateral hemisphere, using the CelLytic MT mammalian tissue lysis/extraction reagent (Sigma-Aldrich, C3228, St. Louis, MO, USA). The antibodies used to detect the blot were rabbit monoclonal anti-alpha Fodrin (EPR3017)-SBDP150 (Abcam, ab75755, Cambridge, UK), monoclonal anti-GFAP (Millipore, MAB360, Billerica, MA, USA), and purified mouse monoclonal antibody Beclin-1 [BD Biosciences, 612113, Fanklin Lakes, NJ, USA; Santa Cruz Biotechnology, Inc., sc-9888, Dallas, TX, USA]. Mouse monoclonal anti-β-actin (Sigma-Aldrich, A5441, St. Louis, MO, USA) served as an internal control. Cell lysates were resolved with 10% sodium dodecyl sulfate-polyacrylamide gel electrophoresis, blotted with the antibodies mentioned above, and incubated with the corresponding secondary antibodies. Proteins were visualized by following the manufacturer’s instructions (Pierce ECL Western blotting substrate, Thermo Scientific, Waltham, MA, USA). The experimenter was blinded to the samples when the protein expression was quantified.

### 4.7. Rotarod Test

To understand the role of ARs in TBI, we used a rotarod device (SINGA Technology Corporation, Taiwan) to test the motor deficits that started two weeks after administering TBI. Pretesting data were evaluated one day before TBI. At the beginning of the rotarod test, animals were handled and trained for three consecutive days on the rotarod for 15 min day^−1^. After training, the data were recorded, and the device was set at an accelerating speed to start at an initial speed of 0 rpm and accelerate to 50 rpm over 300 s. Each mouse performed the trial daily for five minutes, five times, with a minute interval at each setting. Each trial on the rod was terminated when the animal fell off, and the time spent on the rotarod was recorded. Data were averaged and represented for each experimental day.

### 4.8. Immunohistochemistry

Depending on the experimental paradigm, 24 h after the behavioral evaluation, or 21 days after injury, all animals were anesthetized and then transcardially perfused, using heparin followed by 4% formaldehyde. The perfused brains were removed quickly and post-fixed overnight in 4% formaldehyde. The brain tissues were then transferred to 20% sucrose and frozen in OCT gel for sectioning. Each brain was cut into 10 μm coronal sections on a cryostat (Thermo Fisher Scientific, Waltham, MA, USA), and the slides were stored at −80 °C for histological analysis. We evaluated GFAP expression at 21 days after TBI in selected regions of interest (ROIs) from bregma −0.5, −1, −1.5, −2, −2.5, and −3.0 mm. Slides were washed with PBS three times and rinsed in 0.1% Triton-X 100 for 20 min, and then blocked with 1% normal goat serum in PBS with 0.1% Tween 20 (blocking buffer) for 40 min, at room temperature. The sections were then treated with primary mouse monoclonal antibody GFAP (Millipore, MAB360, Billerica, MA, USA) (1:600) and incubated overnight at 4 °C. After rinsing with PBS, the sections were treated with conjugated secondary antibody anti-mouse IgG (H + L) (Alexa Fluor 555 Conjugate #4409) (1:500) for 2 h, at room temperature, in the dark. Slides were then washed in PBS, mounted with Vectashield DAPI (Vector Laboratories, Burlingame, CA, USA), sealed with coverslips, and stored at −80 °C until analysis. A fluorescence microscope system (Zeiss Axiovision) was used to capture the images of the ROIs. The fluorescent intensity and immunoreactive cells within the cortical ROIs were quantified from six different bregma levels.

### 4.9. Thionine Staining

To evaluate tissue loss, we measured the volume of TBI-induced neuronal loss at the injury sites. As mentioned earlier, six coronal sections of bregma levels with 480 μm intervals were taken. Slides were stained by rinsing with distilled water several times and then transferred to 70%, 95%, and 100% ethanol and defatted with xylene. Slides were then rinsed with thionine buffer, followed by 95% ethanol with galactic acid and 95% ethanol for differentiating variables, until the color disappeared, and then in 100% ethanol for dehydration. Xylene was used for the final dehydration step. The slides were covered with a mounting solution and a coverslip for tissue analysis. Photographs were taken by using a Zeiss microscope, and the volume of tissue loss in the TBI-induced injury hemispheres was measured by using Axiovision software. The volume of tissue loss in each bregma level between the two sections was calculated by using the d*(A1 + A2)/2 formula, where d indicates the distance between sections, and A1 and A2 are the measured areas in the two different sections.

### 4.10. Statistical Analysis

Data are presented as mean ± standard error (SEM). One-way ANOVA and one-way ANOVA with repeated measures were used for the data analysis. The paired *t*-test was used to calculate differences between the groups. A *p* < 0.05 was considered statistically significant. All statistical analyses were performed by using StatView 5.0. Bar charts were made by using Sigma Plot 12.0.

## 5. Conclusions

We have identified, for the first time, the regulatory effects of the androgen receptor within the injured brain. In the present study, knockout of the androgen receptor aggravated the brain lesion and dysregulated the expression of markers of autophagy, necrosis, and astrogliosis after TBI. The present study also found that androgen receptor knockout worsens the TBI outcome affecting not only protein regulation but also behavioral and lesion-volume changes after TBI. Our results report an important mechanism for the neuroprotective multi-regulatory role of the androgen receptor. The present study may provide a better understanding of the physiological role of ARs in TBI and the possible key points for the development of novel therapeutics for patients with TBI.

## Data Availability

All data are available from the corresponding author upon reasonable request.

## Sample Availability

Samples are not available from the authors.
